# Diastolic Blood Pressure Rise During Valsalva as a Surrogate for Sympathetic Activation to Identify Functional Hyperadrenergic Postural Tachycardia Syndrome

**DOI:** 10.21203/rs.3.rs-7377025/v1

**Published:** 2025-09-05

**Authors:** Surat Kulapatana, Luis E. Okamoto, Stefano Rigo, Vasile Urechie, Thomas W. Cayton, Ruijing E. Han, Giris Jacob, William D. Dupont, Raffaello Furlan, Italo Biaggioni, André Diedrich

**Affiliations:** Vanderbilt University School of Engineering; Vanderbilt University Medical Center; Humanitas Clinical and Research Center IRCCS; myDoctorAngel Saga; Vanderbilt University Medical Center; Vanderbilt University School of Engineering; Tel Aviv University School of Medicine; Vanderbilt University Medical Center; Humanitas Clinical and Research Center IRCCS; Vanderbilt University Medical Center; Vanderbilt University Medical Center

**Keywords:** POTS, MSNA, Valsalva, Sympathetic, Hyperadrenergic

## Abstract

**Purpose:**

Muscle sympathetic nerve activity (MSNA) is valuable for POTS management, but microneurography is clinically impractical. We investigated whether the Valsalva phase 2 diastolic blood pressure rise (DBP_VM2l_rise_) could be a surrogate for MSNA during the Valsalva and be used to identify hyperadrenergic POTS.

**Methods:**

We included 21 POTS females and 22 healthy females to perform Valsalva and microneurography. MSNA spike rate was obtained using stationary wavelet transformation. The DBP_VM2l_rise_ cut point for hyperadrenergic POTS was optimized by the golden section search with its correlation to phase 2 MSNA spike rate as an objective function. We defined peripheral sympathetic neurovascular transduction (psNVT) as a ratio of DBP_VM2l_rise_ to early phase 2 MSNA increase. We compared Valsalva responses between the identified hyperadrenergic and non-hyperadrenergic POTS.

**Results:**

The DBP_VM2l_rise_ strongly correlated with the Valsalva phase 2 MSNA spike rate percentage change from baseline in healthy (r = 0.874, p < 0.001). The DBP_VM2l_rise_ equal 15 mmHg optimally separated POTS into 7 hyperadrenergic (≥ 15 mmHg, r = 0.902, p = 0.014) and 14 non-hyperadrenergic (< 15 mmHg, r = 0.629, p = 0.021). Although similar MSNA spike rate, the hyperadrenergic group had higher baseline systolic blood pressure (118 ± 10vs105 ± 12 mmHg, p = 0.026), shorter pressure recovery time (1.15 ± 0.75vs2.59 ± 1.17 s, p = 0.005), and higher psNVT (2.60 ± 1.02vs0.58 ± 0.46 mmHg/spike•s^− 1^, p < 0.001) than the non-hyperadrenergic POTS.

**Conclusion:**

DBP_VM2l_rise_ ≥ 15 mmHg could be a marker of hyperadrenergic response to subtype POTS. We defined this subset of POTS as functional hyperadrenergic POTS since they displayed hyperadrenergic phenotypes despite similar level of MSNA to other POTS. The higher psNVT of the hyperadrenergic group suggested a novel pathophysiology of enhanced neurovascular coupling.

## Introduction

Postural tachycardia syndrome (POTS) is a heterogeneous condition associated with a complex pathophysiology. The patients with POTS experience aggravated orthostatic symptoms accompanied with sustained increase of heart rate (HR) at least 30 beats per minute upon standing without orthostatic hypotension^[1]^. In a subset of these patients, the main pathophysiological mechanism has been proposed to be a primary sympathetic activation (Hyperadrenergic POTS). Sympathetic activation can be measured directly by microneurography techniques to obtain muscle sympathetic nerve activity (MSNA). This technique requires high specialized skills, equipment, and is not available in clinics. Indirect clinical markers of increased sympathetic activity like high systolic blood pressure (SBP) overshoot during Valsalva, orthostatic hypertension, high orthostatic plasma catecholamines^[2–5]^, and response to sympatholytic drugs have been proposed^[6, 7]^. Recently, we showed that POTS patients with clinical hyperadrenergic features can be identified by an exaggerated diastolic blood pressure (DBP) increase during late phase 2 of the Valsalva maneuver (DBP_VM2l_rise_) and that this biomarker can also identify those who improved POTS symptoms with central sympatholytic therapy with guanfacine^[8]^.

The Valsalva maneuver is a robust autonomic function test evaluating both sympathetic and parasympathetic cardiovascular responses. A forced expiration against resistance increases intrathoracic pressure and reduces venous return, resulting in a blood pressure (BP) drop that triggers baroreflex-mediated hemodynamic. Thus, the HR and BP responses during Valsalva have been used to derive markers of sympathetic and parasympathetic function. SBP overshoot, which is the highest SBP in Valsalva phase 4 above baseline^[9, 10]^, pressure recovery time (PRT)^[9, 11]^, which measures the duration required for BP returning to baseline after its nadir have been proposed as markers of sympathetic function. The proportion of the BP drop in Valsalva phase 3 over the PRT^[11–13]^ has been defined as adrenergic baroreflex sensitivity (BRSa). Moreover, the DBP_VM2l_rise_, a marker of sympathetic-mediated vasoconstriction, was shown to be able to identify patients with POTS with high resting sympathetic nerve activity (i.e., hyperadrenergic POTS)^[8]^. Given that the number of MSNA spikes and firing rate are increased in response to the BP fall during phase 2 of the Valsalva maneuver^[12, 14]^, this biomarker could provide strong evidence of sympathetic overactivity. However, a direct causal correlation between increased MSNA and increased DBP within the Valsalva maneuver has not been shown and needs to be investigated.

In this study, we hypothesized that the DBP_VM2l_rise_ would be a surrogate for sympathetic function and neurovascular coupling during the Valsalva. We test that a well-defined cut point of diastolic blood pressure rise could be a useful clinical parameter for POTS subtyping. To test this hypothesis, we first identified the optimal cut point value for DBP_VM2l_rise_ to distinguish hyperadrenergic from non-hyperadrenergic POTS using the MSNA-blood pressure relationship. Then, we validated the hyperadrenergic state by symptoms, plasma catecholamines, and Valsalva sympathetic metrics.

## Methods

### Participants

We recruited 23 female patients with POTS and 23 healthy female subjects with age between 18–55 years old. All patients with POTS met the following criteria: 1) heart rate increases at least 30 beats/min within 10 minutes after standing, 2) no orthostatic hypotension, and 3) chronic orthostatic symptoms for at least 6 months. Subjects stopped any medications affecting the autonomic nervous system for at least five half-lives before studies. Minimal number of psychiatric drugs may be continued as necessary, after consulting with a team of physicians. All participants gave their informed consent.

### Protocol

#### Protocol

The study was approved by the Vanderbilt University Institutional Review Board in Human Research and registered at clinicaltrials.gov (NCT04050410). The protocol was conducted at Vanderbilt Autonomic Dysfunction Center.

On the screening day, all subjects performed an active stand test with intermittent HR and BP measurements at 3, 5, and 10 minutes after standing. Orthostatic vitals were chosen from the highest upright HR and an average BP between measurements at 3 and 5 minutes^[15]^. Supine and upright plasma norepinephrine (NE) level were collected during the active stand test. On the study day, after subjects were supine for ≥ 30 minutes before studies to allow fluid redistribution, blood volume was measured by the carbon monoxide rebreathing method. Blood volume deviation (BV_deviation_) in percent to normal values were calculated based on weight, height, and sex^[16]^. The autonomic function tests were started after at least 30 minutes supine. Subjects were asked to breath normally for 5 minutes for baseline recordings. After this, subjects performed a training Valsalva prior to the Valsalva for analysis. In each Valsalva, subjects were asked to relax for 1 minute, then blow into a 10 mL syringe with a tiny leak (22G hole) at a pressure of 30 mmHg for 15 seconds followed by a quiet and relaxing period of one minute.

### Data Recording

Microneurography of the left peroneal nerve was performed using a unipolar tungsten electrode (2 MOhms, FCH, Inc, Bowdoinham, ME). MSNA was filtered (300Hz-5kHz) and amplified (gain range 50μV and resolution 25nV) with stage amplifier (Neuroamp EX, ADInstruments Inc, Colorado Springs CO). Satisfactory recordings of MSNA were defined by 1) heart pulse synchronicity; 2) facilitation during Valsalva straining and suppression during the hypertensive overshoot phase after release; and 3) no changes during tactile or auditory stimulation.

Electrocardiogram (ECG, Ivy 450C, Ivy Biomedical Systems, Inc, Brandford CT, USA), continuous finger blood pressure (Finapres NOVA, The Netherlands), segmental body impedance (BIM, Diefenbach GmbH, Germany), and MSNA were recorded with Powerlab 16/35 and Labchart 8 (ADInstruments Inc, Colorado Springs CO). Used sample rates were 20kHz for MSNA and 1 kHz for ECG and blood pressure. Finger blood pressure values were cross-calibrated with oscillometric brachial blood pressure arm cuff measurements during baseline (Ivy 450C, Ivy Biomedical Systems, Inc, Brandford CT, USA).

### Data processing and analysis

Data were processed with our customized MATLAB software (Physiowave©, A. Diedrich, Vanderbilt University Medical Center, TN, USA). Fiducial points in ECG and continuous blood pressure waveforms were detected and visually verified. Beat-to-beat time series were validated, interpolated, and resampled at 5Hz. Stroke volume (SV), cardiac output (CO), and total peripheral resistance (TPR) were derived from blood pressure waveforms (Modelflow^®^, Finapres Medical System BV, the Netherlands)^[17]^. Baseline values were averaged over the 5-minute resting period. Power spectral analysis was performed using Fast Fourier Transformation (FFT) based Welch’s method^[18]^.

MSNA spikes were denoised and detected by the stationary wavelet transformation with the two-stage kurtosis method^[19]^. Spike rate was estimated by convolving delta functions at spike locations with a 3 Hz cut-off frequency Gaussian filter^[20]^. Additionally, beat-to-beat mean spike rate was calculated by averaging the spike rate for each R-R interval. Burst rate and burst incidence were derived from the integrated MSNA (resistance-capacitance low pass filter with time constant of 0.1s) during resting supine baseline^[21, 22]^.

**Figure 1:**
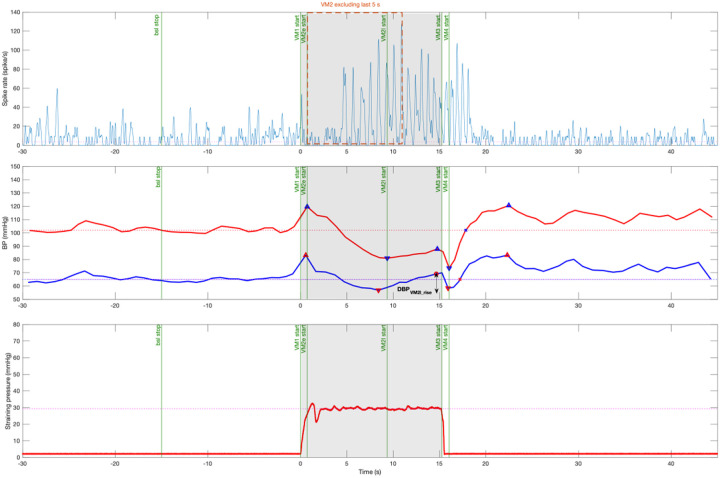
Example recording of a healthy subject performing a 30-mmHg Valsalva maneuver. Valsalva phases were defined based on straining time and blood pressure changes (green vertical lines). Bsl, baseline; VM1, Valsalva phase 1; VM2e, early Valsalva phase 2; VM2l, late Valsalva phase 2; VM3, Valsalva phase 3; VM4, Valsalva phase 4. Top panel: continuous MSNA spike rate smoothed by a 3 Hz cut off frequency Gaussian filter. Orange box represents MSNA spike rate in phase 2 excluding the last 5 seconds. Middle panel: continuous finger systolic blood pressure (SBP, red) and diastolic blood pressure (DBP, blue). Horizontal lines represent baseline blood pressures. The late phase 2 DBP rise (DBP_VM2l_rise_) is the difference between VM2 DBP nadir and following maximum at the end of VM2 (vertical line with arrows). Bottom panel: Valsalva straining pressure. Subject was asked to hold pressure at 30 mmHg for 15 seconds (gray area).

Following common Valsalva maneuver phases were defined and analyzed: Valsalva baseline phase from 45 s to 15 s prior to the straining. Phase 1 (VM1) begins at the onset of straining. Phase 2 (VM2) starts at the maximum blood pressure until the end of the straining. VM2 was divided into early (VM2e) and late (VM2l) phases at the nadir of SBP. Phase 3 (VM3) starts after releasing the strain and ends at minimum blood pressure. Finally, phase 4 (VM4) is the period of blood pressure recovery to baseline level, overshoot, and return to baseline level (Fig. 1). The late phase 2 DBP rise (DBP_VM2l_rise_) is defined as the difference between VM2 DBP nadir and following maximum at the end of VM2.

Top panel: continuous MSNA spike rate smoothed by a 3 Hz cut off frequency Gaussian filter. Orange box represents MSNA spike rate in phase 2 excluding the last 5 seconds.

Middle panel: continuous finger systolic blood pressure (SBP, red) and diastolic blood pressure (DBP, blue). Horizontal lines represent baseline blood pressures. The late phase 2 DBP rise (DBP_VM2l_rise_) is the difference between VM2 DBP nadir and following maximum at the end of VM2 (vertical line with arrows).

Bottom panel: Valsalva straining pressure. Subject was asked to hold pressure at 30 mmHg for 15 seconds (gray area).

MSNA spike rate of each phase was analyzed in absolute value and delta change from Valsalva baseline level. The delta change values were also expressed as percentage change from baseline phase of the Valsalva. When studying the association between MSNA and blood pressure changes in Valsalva phase 2, we excluded the last 5 seconds of MSNA activity to account for the 5 seconds delay on blood pressure response. In other words, MSNA during the last 5 seconds of phase 2 would not affect the blood pressure values at the end of phase 2 (Fig. 1, orange box)^[23]^. We defined central sympathetic baroreflex sensitivity (csBRS) as the ratio between delta change spike rate and preceding delta change diastolic blood pressure within early phase 2 (Eq. 1a). In addition, peripheral sympathetic neurovascular transduction (psNVT) was defined as a ratio between diastolic blood pressure rise during late phase 2 and preceding spike rate change in early phase 2 (Eq. 1b).

Equation 1 central sympathetic baroreflex sensitivity and peripheral sympathetic neurovascular transduction.


(Eq. 1a) $$\text{csBRS}=\frac{\text{mean VM2e spike rate}-\text{mean baseline spike rate}}{\text{mean baseline DBP}-\text{minimum VM2e DBP}}$$

(Eq. 1b) $$\text{psNVT}=\frac{\text{maximum VM2l DBP}-\text{minimum VM2e DBP}}{\text{mean VM2e spike rate}-\text{mean baseline spike rate}}$$

csBRS = central sympathetic baroreflex sensitivity (spike•s^− 1^/mmHg)

psNVT = peripheral sympathetic neurovascular transduction (mmHg/spike•s^− 1^)

VM2e = early phase 2 of Valsalva maneuver

VM2l = late phase 2 of Valsalva maneuver

DBP = diastolic blood pressure (mmHg)

### Cut point search for DBP rise (DBP)

Our primary objective was to find the optimal cut point value for DBP_VM2l_rise_ to distinguish hyperadrenergic from non-hyperadrenergic POTS. We defined the optimal DBP_VM2l_rise_ cut point as the cut point where the highest correlations between MSNA and DBP_VM2l_rise_ were found in both hyperadrenergic (above cut point value) and non-hyperadrenergic POTS (below cut point value) using the golden section search method, as detailed in Appendix 1^[24]^.

### Statistical analysis

Data were presented as mean ± standard deviation (SD) unless otherwise specified. Comparisons between healthy and patients with POTS were performed using unpaired t-test for normally distributed data or the Wilcoxon rank-sum test for non-normally distributed data. Data normality was checked by the Anderson-Darling test. Correlation analyses were done using Pearson’s linear correlation. Statistical analyses were performed in MATLAB (The MathWorks, Inc., Natick, MA). Two tailed p-Values ≤ 0.05 were considered statistically significant.

## Results

### Participants, general characteristics, and normal breathing hemodynamics

One healthy subject and two patients with POTS were excluded due to arrhythmia and poor Valsalva straining quality. In total, twenty-two healthy subjects and twenty-one patients with POTS were included in the analysis of the baseline study. Out of twenty-one patients with POTS, only nineteen subjects had valid MSNA recordings. Patients with POTS and controls were similar in age, weight, and height, but patients with POTS had lower blood volume (Table 1, p < 0.001), higher orthostatic HR increase (p < 0.001), higher supine resting HR (Table 2; p < 0.001), higher SBP (p = 0.011), higher DBP (p = 0.024). POTS had significant lower root mean square of successive differences between heartbeats (RMSSD), a time domain parameter of cardiovagal modulation (p = 0.015). Similarly, the spectral analysis index of cardiovagal modulation (logHF_RRI_) tended to be lower in POTS, but it did not reach statistical significance (P = 0.077). Other spectral analysis parameters, and resting MSNA burst rate and burst incidence were not different between the two groups (Table 2).

### Heart rate, blood pressure, and MSNA spike rate during Valsalva maneuver

**Figure 2:**
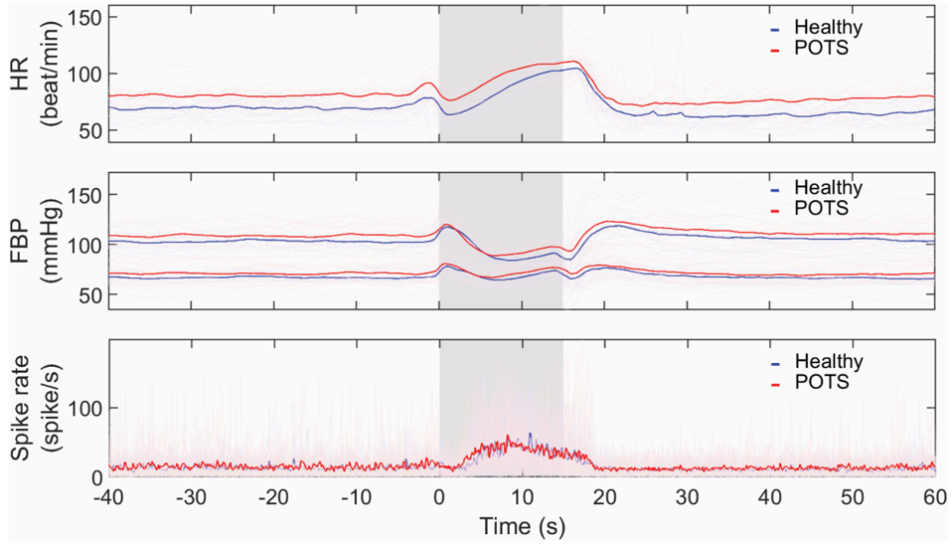
Overlay plots of heart rate (HR, top, healthy n = 22, POTS n = 21), finger blood pressure (FBP, middle, healthy n = 22, POTS n = 21) where upper line represents systolic blood pressure and lower line represents diastolic blood pressure, and MSNA spike rate (bottom, healthy n = 17, POTS n = 19) changes during Valsalva maneuver. Light lines represent individual responses. Dark lines are groups’ average. Gray areas show 30-mmHg Valsalva straining period for 15 seconds.

POTS had significantly higher heart rate than healthy subjects during the baseline (POTS vs healthy, 81 ± 14 vs 70 ± 9 beat/min, p = 0.005) and early phase 2 of Valsalva (101 ± 16 vs 90 ± 17 beat/min, p = 0.041). Blood pressure responses were generally comparable throughout the Valsalva maneuver. MSNA spike rates were not different between the 2 groups both during baseline (14.28 ± 6.38 vs 13.36 ± 6.37 spike/s, p = 0.670) and early phase 2 of Valsalva (32.83 ± 15.41 vs 29.55 ± 11.62 spike/s, p = 0.680) (Fig. 2).

Valsalva sympathetic markers including SBP overshoot (19 ± 11 vs 20 ± 15 mmHg, p = 0.714), PRT (2.11 ± 1.24 vs 3.10 ± 5.62 s, p = 0.932), and DBP_VM2l_rise_ (13 ± 10 vs 13 ± 10 mmHg, p = 0.961) as well as csBRS (2.91 ± 3.91 vs 3.05 ± 5.66 spike•s^− 1^/mmHg, p = 0.590) and psNVT (1.22 ± 1.16 vs 1.07 ± 0.76 mmHg/spike•s^− 1^, p = 0.899) were not different between POTS and healthy. Valsalva ratio was also similar between the two groups (1.86 ± 0.41 vs 1.98 ± 0.32, p = 0.287).

### Correlations between Valsalva phase 2 MSNA and DBP_VM2l_rise_

**Figure 3:**
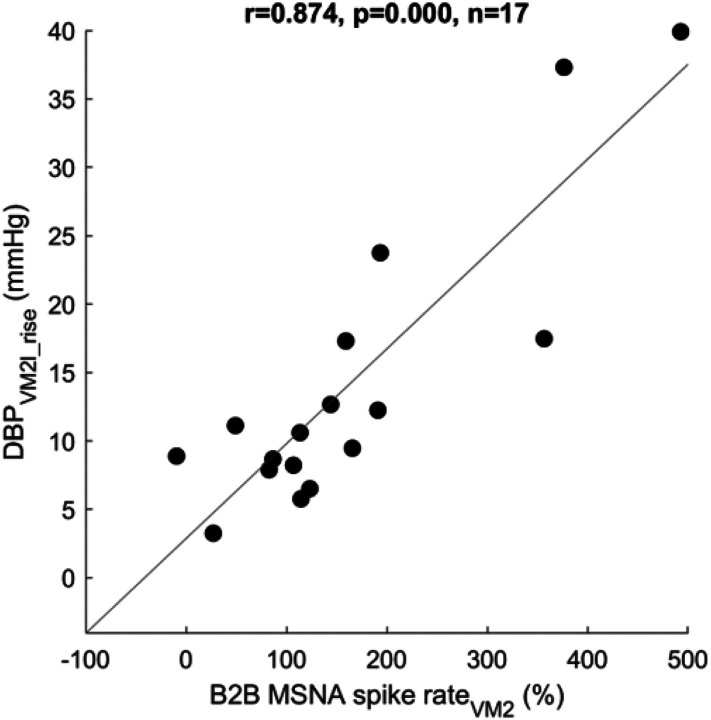
Correlation between beat-to-beat mean spike rate change during phase 2 except last 5 seconds in percent from baseline (B2B MSNA spike rate_VM2_) and diastolic blood pressure rise during late phase 2 (DBP_VM2l_rise_) in healthy control (r=0.874, p<0.001).

**Figure 4:**
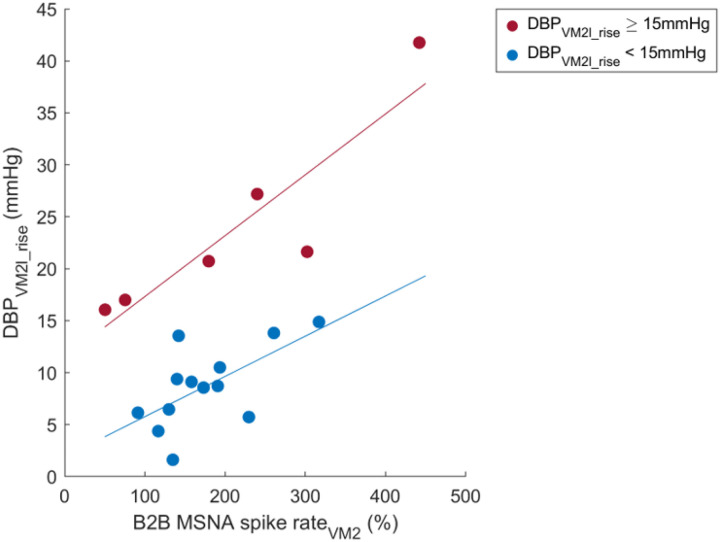
Correlation analyses between beat-to-beat mean MSNA spike rate percentage difference from baseline during phase 2, excluding last 5 s (B2B MSNA spike rate_VM2_) and late phase 2 DBP rise (DBP_VM2l_rise_) in POTS patients having DBP_VM2l_rise_ ≥ 15 mmHg (r=0.902, p=0.014, n=6, red) and in POTS patients having DBP_VM2l_rise_ < 15 mmHg (r=0.629, p=0.021, n=13, blue).

We found a strong correlation between the phase 2 beat-to-beat mean spike rate percentage change from baseline (B2B MSNA spike rate_VM2_) and the DBP_VM2l_rise_ in healthy (r = 0.874, p < 0.001; Fig. 3). The correlation was weaker in POTS (r = 0.661, p = 0.002) due to possible higher heterogeneity (Fig. 4). Correlation between absolute baseline spike rate and DBP_VM2l_rise_ was not significant.

### Golden section search for DBP_VM2l_rise_ optimal cut point

#### Comparison between POTS groups with DBP_VM2l_rise_ ≥ 15 versus < 15 mmHg.

All POTS patients in the DBP_VM2l_rise_ ≥ 15 mmHg group had reported at least one hyperadrenergic symptom on the prescreening questionnaire. The proportion of patients having palpitation, profuse sweating, and flushing episode were 86%, 71%, and 57%, respectively. There were no differences in age, orthostatic BP, orthostatic NE, and blood volume status between POTS groups with DBP_VM2l_rise_ ≥ 15 versus < 15 mmHg (Table 3).

**Figure 5:**
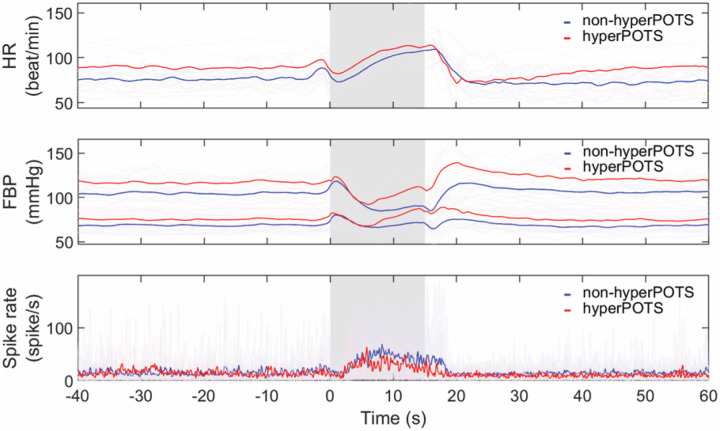
Overlay plots of hyperadrenergic POTS (hyperPOTS, red) and non-hyperadrenergic POTS (nonhyperPOTS, blue) of heart rate (HR, top, non-hyperPOTS n = 14, hyperPOTS n = 7), finger blood pressure (FBP, middle, non-hyperPOTS n = 14, hyperPOTS n = 7) where upper line represents systolic blood pressure and lower line represents diastolic blood pressure, and MSNA spike rate (bottom, non-hyperPOTS n = 13, hyperPOTS n = 6) changes during Valsalva maneuver. Light lines represent individual responses. Dark lines are groups’ average. Gray areas show 30-mmHg Valsalva straining period for 15 seconds.

At baseline phase of Valsalva, the POTS group with DBP_VM2l_rise_ ≥ 15 mmHg (n = 7) had higher SBP (118 ± 10 vs 105 ± 12 mmHg, p = 0.026) and HR (90 ± 19 vs 76 ± 10 beat/min, p = 0.038) than those with DBP_VM2l_rise_ <15 mmHg (n = 14). The high DBP_VM2l_rise_ group also had higher systolic blood pressure than the other POTS patients throughout the end of late phase 2 (116 ± 17 vs 93 ± 15 mmHg, p = 0.005), and peak of phase 4 (143 ± 21 vs 120 ± 17 mmHg, p = 0.019) of the Valsalva, while the SBP drop in early phase 2 were similar (−27 ± 9 vs −22 ± 9 mmHg, 0.200). Interestingly, the duration of early phase 2 (point of SBP nadir) was shorter in the DBP_VM2l_rise_ ≥ 15 mmHg group (5.57 ± 1.93 vs 8.28 ± 2.59 s, p = 0.025) (Fig. 5).

Absolute MSNA spike rate of both groups were not different during baseline (13.45 ± 7.08 vs 14.66 ± 6.30 spike/s, p = 0.714) and early phase 2 (24.83 ± 7.23 vs 36.52 ± 16.97 spike/s, p = 0.127) (Fig. 5). However, the high DBP_VM2l_rise_ group had greater BP responses with respected to the activated MSNA spike rate as can be observed by higher psNVT than the other group (2.60 ± 1.02 vs 0.58 ± 0.46 mmHg/spike•s^− 1^, p < 0.001), while the csBRS was not significantly different (0.62 ± 2.69 vs 3.96 ± 4.01 spike•s^− 1^/mmHg, p = 0.084). The PRT was lower (1.15 ± 0.75 vs 2.59 ± 1.17 s, p = 0.005) and the SBP overshoot tended to be higher (25 ± 12 vs 15 ± 10 mmHg, p = 0.079) in the high DBP_VM2l_rise_ group.

### Correlation between DBP_VM2l_rise_ and selected sympathetic-related markers in POTS

**Figure 6:**
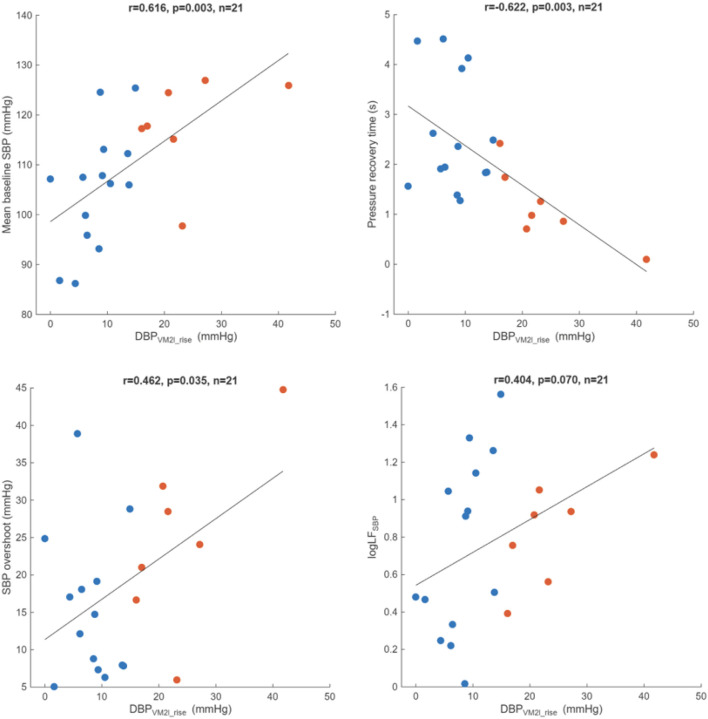
Correlation analyses between late phase 2 DBP rise (DBP_VM2l_rise_) and sympathetic-related markers including mean baseline SBP (upper left, r=0.616, p=0.003), pressure recovery time (upper right, r=−0.622, p=0.003), SBP overshoot (lower left, r=0.462, p=0.035), and logarithm of low frequency power of resting SBP variability (logLF_SBP_, lower right, r=0.404, p=0.070) in POTS having DBP_VM2l_rise_ < 15 mmHg (blue) and ≥ 15 mmHg (red).

DBP_VM2l_rise_ was correlated significantly with Valsalva baseline SBP (r = 0.616, p = 0.003), pressure recovery time (PRT, r=−0.622, p = 0.003), SBP overshoot (r = 0.462, p = 0.035). DBP_VM2l_rise_ did not correlate with the low frequency power of SBP during resting (logLF_SBP_, r = 0.404, p = 0.070, Fig. 6). No correlation was found between the DBP_VM2l_rise_ and blood volume deviation (r=−0.280, p = 0.246).

## Discussion

The current study proposes a DBP_VM2l_rise_ as a clinical marker of peripheral sympathetic function for subtyping hyperadrenergic POTS. The strong correlation between the change of MSNA spike rate in percent baseline and the DBP rise during Valsalva maneuver supports the idea to use the DBP_VM2l_rise_ as a surrogate for sympathetic activation. Valsalva maneuver is a robust autonomic function test examining both sympathetic and parasympathetic functions. Sympathetic responses can be observed from blood pressure increases during late phase 2 and phase 4 of this maneuver. Several sympathetic markers have been derived from phase 4, including SBP overshoot and PRT^[25]^. However, phase 4 blood pressure responses are not exclusively due to vascular α-adrenergic function. They also depend on cardiac β-adrenergic function and increased venous return. We focused on the late phase 2 BP change since it is more proximate to the sympathetic vascular function and vasoconstriction than phase 4^[9, 25–27]^.

A strong correlation between DBP_VM2l_rise_ and the phase 2 MSNA spike rate percentage difference from baseline in healthy subjects warrants the use of DBP_VM2l_rise_ as a sympathetic marker. Wavelet-based MSNA processing provides information on the number of action potentials (MSNA spikes) instead of the conventional number of bursts, which may be inadequate for short autonomic tests like the 15 seconds of the Valsalva phase 2. Although the DBP_VM2l_rise_ is correlated with MSNA percentage changes, its correlation to the actual phase 2 MSNA spike rate is insignificant. These results suggest that vasoconstriction responses to the relative change of MSNA, rather than the absolute neural activity. Calculating MSNA percentage change could standardize the interindividual variation nature of the MSNA that makes it uncorrelated with baseline BP and HR^[28–30]^. Our findings correspond to the previous work by Schrezenmaier et al., which showed a strong correlation between the percentage change of MSNA in early phase 2 and the SBP change in late phase 2^[11]^. The strong correlation of B2B MSNA spike rate_VM2,_ which discards the last 5-second of phase 2 MSNA to account for the rise of late phase 2 DBP, supports a theory of 5-second feedforward delay of the neurovascular response^[23, 31]^. We decided to use average beat-to-beat spike rate rather the simple mean spike rate because of the pulse synchronicity nature of the MSNA firing^[29]^.

We found that DBP_VM2l_rise_ at or over 15 mmHg was an optimal cut point to distinguish hyperadrenergic POTS from other non-hyperadrenergic subtypes. Currently, there are no standard criteria to subtype hyperadrenergic POTS, but we warrant our selection by sympathetic phenotypes in symptoms and other Valsalva metrics. All identified hyperadrenergic POTS have a history suggestive of hyperadrenergic episodes including palpitation, flushing, and excessive sweating. Their average upright NE was over 600 pg/mL (Table 3), but we found no differences in NE levels between hyperadrenergic and non-hyperadrenergic POTS subgroups. The similar NE level does not contradict our assumption about hyperadrenergic state, since the plasma NE concentration depends on both spillover and clearance^[32, 33]^. Another possibility is the fact that plasma NE is not only a marker of a primary hyperadrenergic state but can also be increased by secondary compensatory sympathetic responses. We found that the hyperadrenergic group has higher blood pressure and shorter PRT than the non-hyperadrenergic group. Both high blood pressure and short PRT as well as a trend of higher SBP overshoot in the hyperadrenergic group demonstrate high sympathetic function.

We found no differences in both resting MSNA and MSNA changes during Valsalva between hyperadrenergic POTS and other POTS, which may be deviate to earlier assumption that hyperadrenergic POTS probably have high MSNA. However, we found that the identified hyperadrenergic by DBP_VM2l_rise_ showed hyperadrenergic state in symptoms, vital signs, catecholamines, and Valsalva responses. We are proposing a novel pathophysiology of hyperadrenergic POTS in which they may have enhanced neurovascular coupling, instead of having primary high sympathetic tone. These patients could generate higher Valsalva DBP response and have high sympathetic features despite the same level of MSNA activation, compared to other patients with POTS. We may classify this subset of hyperadrenergic POTS as functional hyperadrenergic POTS. The proposed mechanism is confirmed by the higher peripheral sympathetic neurovascular transduction (psNVT) found in the hyperadrenergic group. Furthermore, the hyperadrenergic group has shorter early phase 2 implying that vasoconstrictive response may be faster or more efficient, corresponding to the high psNVT.

The use of DBP_VM2l_rise_ for POTS subtyping, proposed in our previous study, linked the DBP_VM2l_rise_ to baseline MSNA^[8]^. We strengthened our previous work by correlating the DBP_VM2l_rise_ directly to the MSNA during phase 2 of the same Valsalva maneuver. We chose the nonlinear optimization using the golden section search method because it does not require derivation and provides good convergence. The search converges in just 13 iterations and suggests that the 15-mmHg DBP_VM2l_rise_ is the best cut point to separate POTS into 2 groups based on the correlations with the B2B MSNA spike rate_VM2_ (Fig. 4). With this DBP_VM2l_rise_ criterion, we identify 32% (6 out of 19) hyperadrenergic POTS in this patient cohort. The percentage is interestingly close to our previous study in which hyperadrenergic POTS was defined using the 4th quartile of baseline MSNA^[8]^. We may imply that the prevalence of hyperadrenergic POTS could be around 30%. In addition, the DBP_VM2l_rise_ showed its potential to subtyping hyperadrenergic POTS both from 2 difference approaches 1) baseline MSNA with a receiver operating characteristic (ROC) curve from the previous study^[8]^ and 2) Valsalva MSNA with a golden section search optimization from the current study. We could confirm the warrant of the DBP_VM2l_rise_ as a sympathetic noninvasive clinical marker.

Thus, in both our previous and the current report, we confirmed that the increase in diastolic blood pressure during phase 2 of the VM identifies POTS patients with a clinical hyperadrenergic phenotype. However, this clinical biomarker (DBP_VM2l_rise_) is associated either with an increase in resting MSNA^[8]^ or an enhanced neurovascular coupling, as determined by the higher psNVT in the high DBP_VM2l_rise_ group. The significant correlations between DBP_VM2l_rise_ and baseline SBP, PRT, and SBP overshoot confirm the validity of using the DBP_VM2l_rise_ as a sympathetic surrogate. On the other hand, Valsalva responses between healthy and POTS subjects are generally comparable except for the expected higher heart rate in POTS (Fig. 2). A possible explanation could be the heterogeneous pathophysiology of POTS that blurs the differences. Thus, the application of the Valsalva maneuver should be in POTS subtyping rather than POTS diagnosis.

Hyperadrenergic POTS is one of the major subtypes that could benefit from sympatholytics, if correctly identified. Our preliminary results suggest that moxonidine could selectively suppress sympathetic Valsalva responses including the DBP_VM2l_rise_ in hyperadrenergic POTS, even with limited sample size (Appendix 2). These emphasize the importance of subtyping with clinical markers. The central sympatholytic drug, e.g., guanfacine, has been shown to improve symptoms in hyperadrenergic POTS^[8]^. Moreover, blood volume deficit is a common pathophysiology in POTS^[16]^. We found that our defined functional hyperadrenergic POTS and other POTS patients have similar degrees of blood volume deficit (Table 3). The hypovolemia does not argue against our subtyping of hyperadrenergic POTS and the high blood pressure found on them, since there is an inverse relationship between blood volume and blood pressure in orthostatic intolerance including hyperadrenergic POTS patients^[34]^. Chronic use of sympatholytics may improve volume deficit in this patient group since sympathetic overactivity could lead to volume depletion. A clinical trial of moxonidine with larger sample size and longer duration would be needed to clarify these associations.

### Limitations

We anticipated that our work could promote further studies to standardize POTS subtyping. Our identification of an optimal cut point between hyperadrenergic and non-hyperadrenergic POTS patients was based on an exploratory post hoc analysis, which should be confirmed by subsequent studies. Also, the neurovascular transduction or psNVT in our study was estimated by average response between MSNA and DBP during Valsalva. Additional research applying control theory and transfer function analysis between sympathetic activity and blood pressure is needed.

## Conclusion

We proposed a clinical marker from Valsalva maneuver, namely DBP_VM2l_rise_, as a surrogate for sympathetic function applicable for POTS subtyping. The relationship of DBP_VM2l_rise_ to sympathetic function has been confirmed by a strong correlation to B2B MSNA spike rate_VM2_ in healthy subjects. We found, by the golden section search method, that the DBP_VM2l_rise_ ≥ 15 mmHg is an optimal cut-point to subtype hyperadrenergic POTS. The identified hyperadrenergic POTS patients show high sympathetic features in symptoms, plasma NE, and other Valsalva metrics such as high Valsalva baseline SBP and short PRT, but similar MSNA activation to the non-hyperadrenergic group. Thus, we may define them as functional hyperadrenergic POTS. The hyperadrenergic responses in these POTS are possibly due to improved neurovascular coupling as suggested by higher psNVT, compared to POTS with low DBP_VM2l_rise_. The current study enabled a practical clinical marker for POTS subtyping from Valsalva maneuver and hinted a novel pathophysiology for hyperadrenergic POTS, that would allow personalized treatment for them. The mechanisms of functional hyperadrenergic POTS need to be elaborated in further studies.

## Supplementary Material

This is a list of supplementary files associated with this preprint. Click to download.

- 20250811vmhyperpotsappendix1.docx
- 20250811vmhyperpotsappendix2.docx

## Figures and Tables

**Table 1:** General characteristics and blood volume status in female healthy and patients with POTS

	Healthy	POTS	p-value
General characteristics	(n = 22)	(n = 21)	
Age (year)	32 ± 9	28 ± 7	0.559
Weight (kg)	63.4 ± 9.0	67.9 ± 11.0	0.302
Height (cm)	166 ± 7	169 ± 6	0.236
Orthostatic vitals	(n = 21)	(n = 21)	
deltaHR (beat/min)	26 ± 15	45 ± 19	<0.001
deltaSBP (mmHg)	2 ± 9	5 ± 7	0.171
deltaDBP (mmHg)	7 ± 7	10 ± 8	0.162
Orthostatic NE	(n = 22)	(n = 19)	
Supine NE (pg/mL)	217 ± 97	248 ± 123	0.367
Upright NE (pg/mL)	478 ± 243 (n = 20)	578 ± 318	0.361
Upright NE : Supine NE	2.27 ± 0.81 (n = 20)	2.51 ± 1.05	0.436
Blood volume measurements	(n = 16)	(n = 19)	
Hematocrit (Hct, %)	37.32 ± 2.17	38.87 ± 1.98	0.034
Hemoglobin concentration (Hb, g/dL)	12.41 ± 1.11	13.04 ± 0.65	0.071
Blood volume (BV, mL/kg)	74.84 ± 9.12	64.55 ± 10.34	0.004
Blood volume deviation (BV_deviation_, %)	−1.40 ± 10.10	−14.40 ± 10.74	<0.001

Values presented as mean ± SD, BV_deviation_ is a percentage difference between measured blood volume and normal blood volume predicted from sex, weight, and height^[16]^. Orthostatic vitals presented as standing minus supine heart rate difference (deltaHR), systolic blood pressure difference (deltaSBP), and diastolic blood pressure difference (deltaDBP). NE, plasma norepinephrine concentration.

**Table 2:** Spectral powers and hemodynamics of 5-minute supine normal breathing in female healthy and patients with POTS

	Healthy (n = 22)	POTS (n = 21)	p-value
HR (beat/min)	70 ± 9	85 ± 15	<0.001
SBP (mmHg)	104 ± 9	113 ± 12	0.011
DBP (mmHg)	67 ± 6	71 ± 8	0.027
SV (mL)	85 ± 16 (n = 21)	86 ± 13 (n = 21)	0.808
CO (L/min)	5.83 ± 1.08 (n = 21)	7.21 ± 1.45 (n = 21)	0.001
TPR (dyn s/cm^5^)	1176 ± 278 (n = 21)	1003 ± 189 (n = 21)	0.029
RMSSD (ms)	47 ± 25	33 ± 33	0.015
logLF_RRI_	2.86 ± 0.43	2.70 ± 0.40	0.208
logHF_RRI_	2.72 ± 0.47	2.40 ± 0.67	0.077
logLF_SBP_	0.57 ± 0.35	0.78 ± 0.42	0.089
MSNA burst rate (burst/min)	11.05 ± 6.56 (n = 16)	11.49 ± 6.16 (n = 19)	0.838
MSNA burst incidence (burst/100beats)	16.07 ± 10.48 (n = 16)	14.00 ± 8.38 (n = 19)	0.519

Values presented as mean ± SD; HR, heart rate; SBP, systolic blood pressure; DBP, diastolic blood pressure; SV, stroke volume; CO, cardiac output; TPR, total peripheral resistance; RMSSD, root mean square of successive differences between normal heartbeats; LF, low frequency power; HF, high frequency power; RRI, R-R interval of an electrocardiogram; log, base 10 logarithm; MSNA, muscle sympathetic nerve activity.

**Table 3:** General characteristics of POTS having DBP_VM2l_rise_ < 15 mmHg and ≥ 15 mmHg

	POTS DBP_VM2l_rise_ < 15 (n = 14)	POTS DBP_VM2l_rise_ ≥ 15 (n = 7)	p-value
General characteristics			
Age (year)	28 ± 8	29 ± 7	0.921
Weight (kg)	65 ± 7	75 ± 14	0.045
Orthostatic vitals			
deltaHR (beat/min)	51 ± 19	33 ± 12	0.041
deltaSBP (mmHg)	5 ± 6	4 ± 9	0.703
deltaDBP (mmHg)	9 ± 7	12 ± 10	0.555
Orthostatic NE			
Supine NE (pg/mL)	261 ± 134 (n = 12)	227 ± 107	0.570
Upright NE (pg/mL)	550 ± 307 (n = 12)	626 ± 354	0.629
Upright NE : Supine NE	2.39 ± 1.07 (n = 12)	2.72 ± 1.07	0.518
Blood volume measurements			
Blood volume deviation (BV_deviation_, %)	−13.58 ± 10.37 (n = 12)	−15.80 ± 12.05	0.773

Values presented as mean ± SD, BV_deviation_ is a percentage difference between measured blood volume and normal blood volume predicted from sex, weight, and height^[16]^; Orthostatic vitals presented as standing minus supine heart rate difference (deltaHR), systolic blood pressure difference (deltaSBP), and diastolic blood pressure difference (deltaDBP); NE, plasma norepinephrine concentration.
